# The role of the nervous system in the occurrence, development, and immune response of renal cell carcinoma

**DOI:** 10.3389/fimmu.2025.1681503

**Published:** 2025-10-09

**Authors:** Honghong Sun, Lu Liu, Yuchen Mao, Zhongyu Tan, Xuwen Li, Guangyao Li

**Affiliations:** ^1^ Department of Urology, Linfen Central Hospital, Linfen, Shanxi, China; ^2^ First Clinical Medical College, Nanchang University, Nanchang, China; ^3^ Department of Urology, National Cancer Center/National Clinical Research Center for Cancer/Cancer Hospital Chinese Academy of Medical Sciences and Peking Union Medical College, Beijing, China

**Keywords:** renal cell carcinoma, neuroimmune microenvironment, nervous system, tumor-associated macrophages, immunotherapy

## Abstract

Renal cell carcinoma (RCC) represents one of the fastest-growing urological malignancies globally, with approximately 30% of patients presenting with metastatic disease at diagnosis. Despite advances in targeted therapy and immune checkpoint inhibitors, treatment resistance remains a critical challenge, largely attributed to the heterogeneous tumor microenvironment (TME). This review systematically examines the emerging role of the neuroimmune system in RCC pathogenesis and progression. The kidney receives dual innervation from sympathetic and parasympathetic systems, which undergo pathological remodeling during tumorigenesis. Novel findings from RCC preclinical models reveal that MANF protein drives sunitinib resistance via IRE1α-XBP1 pathway inhibition, while NPTX2 aberrantly activates PI3K-Akt survival signaling in clear cell renal cell carcinoma (ccRCC). Therapeutic strategies targeting the neuroimmune axis show promise, including β-blockers combined with PD-1 inhibitors to reverse T cell exhaustion, CXCR4 antagonists disrupting nerve- tumor-associated macrophage (TAM) crosstalk, and radiofrequency ablation of perirenal nerve plexus. Future directions involve spatial transcriptomics mapping of the neuroimmune landscape, developing neurotransmitter-targeted delivery systems, and optimizing sequential combination therapies. Understanding the tripartite interaction between nerves, immune cells, and tumor cells opens new avenues for precision medicine in RCC, potentially establishing neuroimmune modulation as a potential new direction of RCC therapy distinct from anti-angiogenesis and immunotherapy.

## 1 Introduction

Renal cell carcinoma (RCC), one of the fastest-growing urological malignancies in terms of occurrence rate worldwide, accounts for over 400,000 new cases annually (1). Although surgical resection is effective for early-stage patients, approximately 30% of cases are diagnosed at a metastatic stage, with a 5-year survival rate of less than 15% for advanced-stage patients (2, 3). More critically, resistance to targeted therapies (e.g., VEGF inhibitors) and immune checkpoint blockers (e.g., anti-PD-1/PD-L1 agents) has become increasingly prominent — 40% of patients with clear cell renal cell carcinoma (ccRCC) fail to respond to combination therapies, a phenomenon fundamentally linked to the high heterogeneity of the Tumor Microenvironment (TME) (4). This pressing reality underscores the urgent need to elucidate novel mechanisms underlying RCC pathogenesis and progression, thereby providing new insights to overcome current therapeutic limitations.

Conventional viewpoints hold that the nervous system — which arises as the regulatory hub during the third week of embryonic development — primarily mediates the coordination of organ function through neurotransmitters such as norepinephrine and acetylcholine. However, groundbreaking advances in tumor neurobiology over the past decade have fundamentally challenged this notion. In prostate cancer, a 2.5-fold increase in sympathetic nerve density activates β2-adrenergic receptors (ADRB2), driving angiogenesis and metastasis via the cAMP-PKA signaling pathway (5). Similarly, breast cancer cells hijack neuronal mitochondria through tunneling nanotubes (TNTs), augmenting oxidative phosphorylation to facilitate brain metastasis (5). These findings unequivocally demonstrate that the nervous system serves as a pivotal architect of the tumor microenvironment, thereby establishing a novel framework for investigating neural regulatory mechanisms in RCC.

The kidneys, as an organ dually innervated by the sympathetic nerves (T7-L1 spinal segments) and parasympathetic nerves (vagus nerve) (6), exhibits remarkable regional specificity in its neural distribution: The renal hilum is densely populated with sympathetic nerve fibers that regulate renin release and vascular tone; the peritubular cortical regions are richly innervated by sensory nerve endings that detect injury signals; while the intrinsic neuronal network maintains tissue homeostasis through the secretion of neurotrophic factors such as GDNF and NGF (7). During RCC progression, this neural network undergoes malignant remodeling: Clinicopathological studies have confirmed that the synaptic protein NPTX2 (which is not expressed in healthy kidneys) is aberrantly overexpressed in ccRCC, where it activates the PI3K-Akt pro-survival pathway via binding to the AMPA receptor GluA4 (8). Concurrently, MANF protein drives sunitinib resistance in VHL-deficient tumors by suppressing the IRE1α-XBP1 endoplasmic reticulum stress pathway (9). These findings demonstrate that the nervous system does not merely respond passively to tumors but actively participates in the malignant evolution of RCC.

The tumor immune microenvironment of RCC exhibits a notable paradox: Phenotypic analysis of tumor-infiltrating and peripheral blood T cells from patients reveals that despite dense CD8^+^ T cell infiltration, these cells predominantly co-express immune checkpoint markers such as PD-1^+^TIM-3^+^, indicating a state of profound functional exhaustion (10). Recent studies have identified the nervous system as the central regulator of this paradox through three distinct mechanisms: Firstly, sympathetic-driven immunosuppression: Norepinephrine (NE) released by sympathetic nerves is taken up by TAMs expressing norepinephrine transporters (NETs). This activates the β2-AR-cAMP pathway, promoting IL-10 secretion while suppressing T cell activity (11). Secondly, dysregulated cholinergic anti-inflammatory signaling: Reduced acetylcholine release from parasympathetic nerves in RCC leads to attenuated α7nAChR signaling, failing to suppress NF-κB-mediated proinflammatory cytokine storms (12). Lastly, hyperactivated neuropeptide-immune metabolic axis: Substance P released from sensory nerves activates CD39^+^CD73^+^ regulatory T cells (Tregs) via neurokinin 1 receptor (NK1R), catalyzing ATP hydrolysis to adenosine. This subsequently activates A2AR receptors to block dendritic cell (DC) antigen presentation (13). These three mechanisms synergistically shape RCC’s unique “high-infiltration/low-response” immune landscape.

This review aims to systematically analyze the dynamic patterns of neural innervation and reveal the evolution of nerve density in the occurrence and metastasis of RCC; Elucidate the molecular mechanisms of neurotransmitters (NE/ACh), neuropeptides (SP/VIP), etc., and elucidate their tumor immunity mediated by ADRB/α7nAChR/NK1R receptors; New therapeutic strategies targeting the neuroimmune axis, such as using propranolol (ADRB2 antagonist) in combination with PD-1 inhibitors to reverse T cell depletion, or blocking drug resistance pathways with GDNF antibodies, provide new pathways for clinical intervention.

## 2 Innervation of the kidney

**Figure 1:**
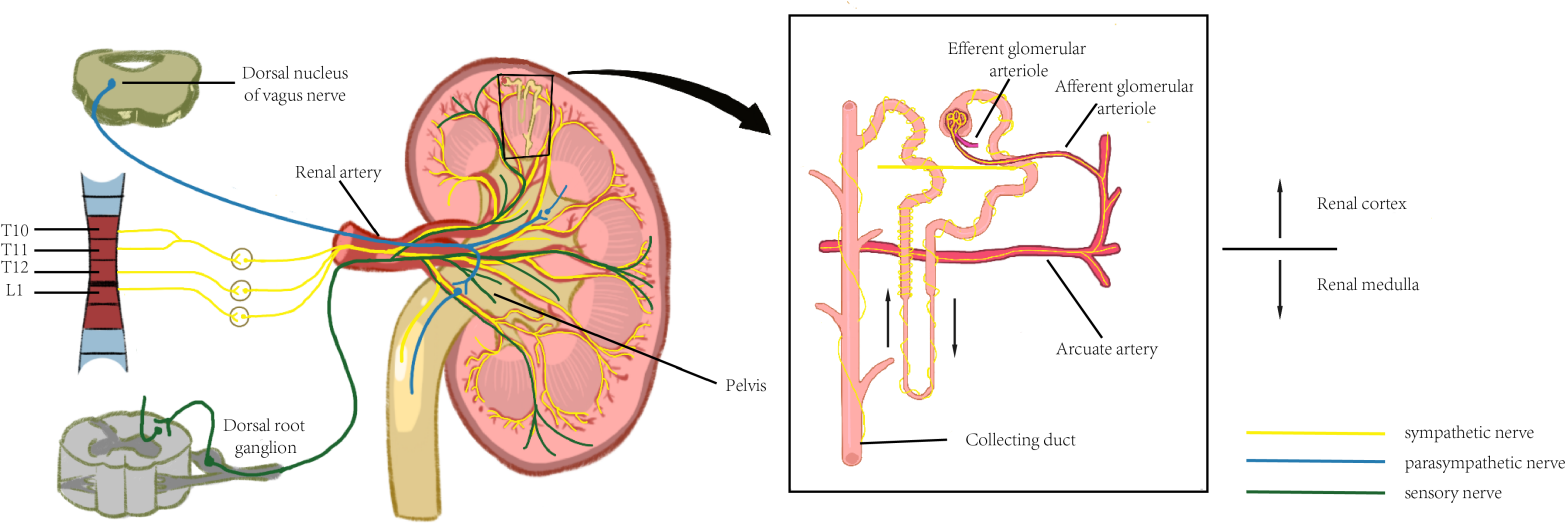
The neural innervation of kidney. Significant regional variations can be observed with the distribution of the renal nervous system, and this anatomical feature enables the nervous system to efficiently control renal function and maintain renal health.

The kidney is dually innervated by sympathetic and parasympathetic nerves (6), primarily originating from the renal plexus, with visceral sensory nerves also distributed. Among these, the sympathetic nerves serve as the predominant innervating fibers, while parasympathetic nerves are exclusively distributed to the smooth muscle of the renal pelvis. (**Figure 1**).

### 2.1 Sympathetic and parasympathetic innervation

The kidney is subjected to sympathetic nerve fiber innervation originating from the thoracic (T6–T12) and lumbar (L1) segments of the spinal cord. The sympathetic nerves reach the afferent and efferent arterioles of the renal cortex via the renal artery and its subsequent vascular branches, with the highest density of tyrosine hydroxylase (TH)-positive nerve fibers observed at the afferent arterioles. Fine vasculature may also receive sympathetic innervation, though at a lower density than that of arterioles. No significant difference in sympathetic innervation is observed between the vasculature associated with superficial and deep nephrons. Tubular innervation is less dense than that of the vasculature, with the thick ascending limb exhibiting the highest density of nerve fibers, while the collecting duct demonstrates relatively sparse innervation compared to other structures (14). The renal pelvis is less densely innervated by sympathetic fibers than the vasculature, with sympathetic and sensory fibers distributed separately within it. The neural pathway from the brain to the kidneys follows a pattern similar to that of other visceral organs. The renal sympathetic innervation follows a two-neuron pathway: the cell body of the first-order (preganglionic) neuron is located in the intermediolateral (IML) cell column of the spinal cord (T1–L2). This preganglionic neuron forms synapses with postganglionic neurons in either paravertebral ganglia (i.e., within the sympathetic trunk) or prevertebral ganglia (e.g., celiac, superior mesenteric, or aorticorenal ganglia). The postganglionic fibers then travel along the renal vasculature to the kidneys. The cell bodies of preganglionic parasympathetic neurons innervating the kidneys are located in the dorsal motor nucleus of the vagus nerve. These fibers join the celiac and renal plexuses via the vagus nerve, with postganglionic fibers originating from the aorticorenal ganglia. These fibers distribute along the renal vasculature, primarily terminating in the smooth muscle of the renal pelvis, inducing renal vasodilation and pelvic contraction.

### 2.2 Sensory innervation

The kidney is also richly innervated by sensory nerve fibers, whose cell bodies are located in the dorsal root ganglion (DRG) and are responsible for detecting local injury and inflammatory signals. The renal vasculature is endowed with sensory innervation, whose density varies across all branches of the renal artery: the distal arterial branches possess the highest innervation density, whereas sensory fibers are scarcely associated with afferent or efferent arterioles and are absent from glomeruli. The renal veins also receive sparse innervation. Recent studies suggest that sensory innervation in the renal cortex may be more prominent than previously recognized, particularly regarding the association of sensory fibers with afferent and efferent arterioles and potentially with glomeruli. Sensory innervation of renal tubules is minimal, whereas the renal pelvis exhibits the highest density of sensory nerve fibers among the kidney’s structural components. The renal sensory nerves originate from small- to medium-diameter neuronal cell bodies located in the DRG of the lower thoracic and upper lumbar spinal cord.

### 2.3 Physiological functions of renal innervation

Sympathetic nerve fibers release NE, adenosine triphosphate (ATP), neuropeptide Y (NPY), and vasoactive intestinal peptide (VIP) in the kidney, among which NE exerts the most significant influence on renal function via its action on adrenergic receptors. By releasing NE, sympathetic nerves act on α1- and β-adrenergic receptors located on renal tubular epithelial cells and vascular smooth muscle cells, thereby regulating sodium reabsorption and renin release. Studies have demonstrated that NE can also directly activate sodium-glucose cotransporter 2 (SGLT2) expression in proximal tubular epithelial cells, promoting glucose reabsorption—a mechanism of critical importance in diabetic nephropathy and metabolic reprogramming in RCC (15).

Upon renal injury or inflammation, sensory nerves are activated, transmitting signals to the central nervous system (CNS), particularly to brain regions such as the subfornical organ (SFO) and paraventricular nucleus (PVN). This afferent signaling triggers a neural reflex that further activates efferent sympathetic pathways, forming a kidney-brain-kidney sympathetic reflex arc (16). In chronic kidney disease (CKD) models, sustained activation of this reflex arc elevates sympathetic tone, which in turn exacerbates renal inflammation and fibrosis, thereby perpetuating a vicious cycle.

The distribution of renal nerves exhibits marked regional heterogeneity: nerve density is significantly higher in the cortical region than in the medulla, with the juxtaglomerular apparatus—a key regulatory site of the renin-angiotensin-aldosterone system (RAAS)—being particularly densely innervated. This anatomical feature enables the nervous system to efficiently modulate glomerular filtration rate, renal blood flow, and tubular reabsorption functions.

## 3 Neural mediators and their mechanisms of action in the renal immune microenvironment

**Figure 2:**
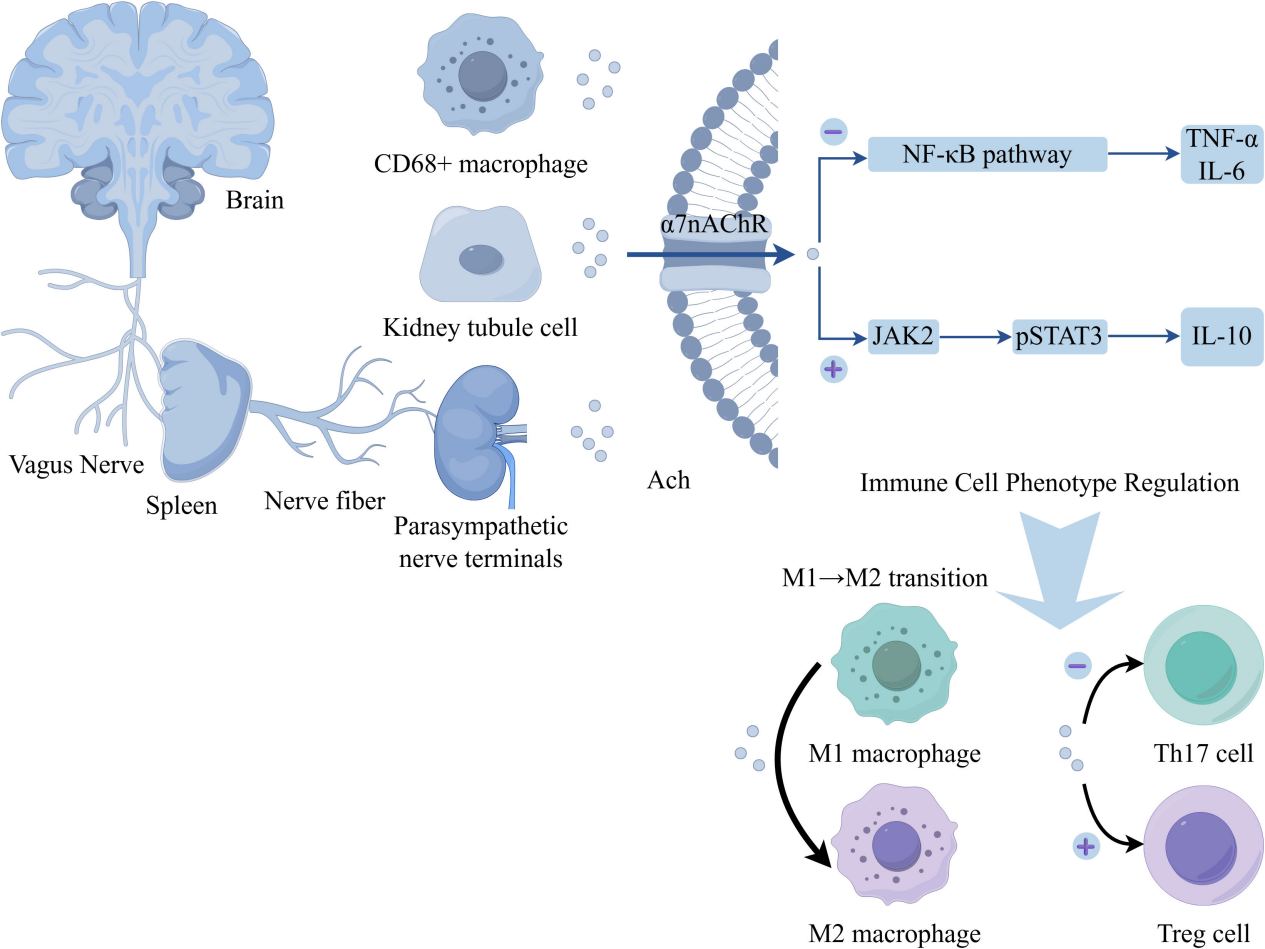
Schematic depiction of the vagus nerve‐mediated cholinergic modulation of immune cell phenotypes. The vagus nerve connects the brain to the spleen and extends parasympathetic nerve terminals near renal tubule cells. Acetylcholine (Ach) acts on the α7 nicotinic acetylcholine receptor (α7nAChR) expressed on cells such as CD68^+^ macrophages and renal tubule cells. α7nAChR activation exerts dual effects on signaling pathways: (1) Inhibiting the NF‐κB pathway, thereby reducing production of pro‐inflammatory cytokines (TNF‐α, IL‐6); (2) Activating the JAK2–pSTAT3 pathway, which promotes synthesis of the anti‐inflammatory cytokine IL‐10. These signaling events drive functional reprogramming of immune cells: facilitating the M1→M2 macrophage transition, suppressing Th17 cell differentiation (negative regulation, “⊖”), and promoting regulatory T cell (Treg) development (positive regulation, “⊕”).

**Figure 3:**
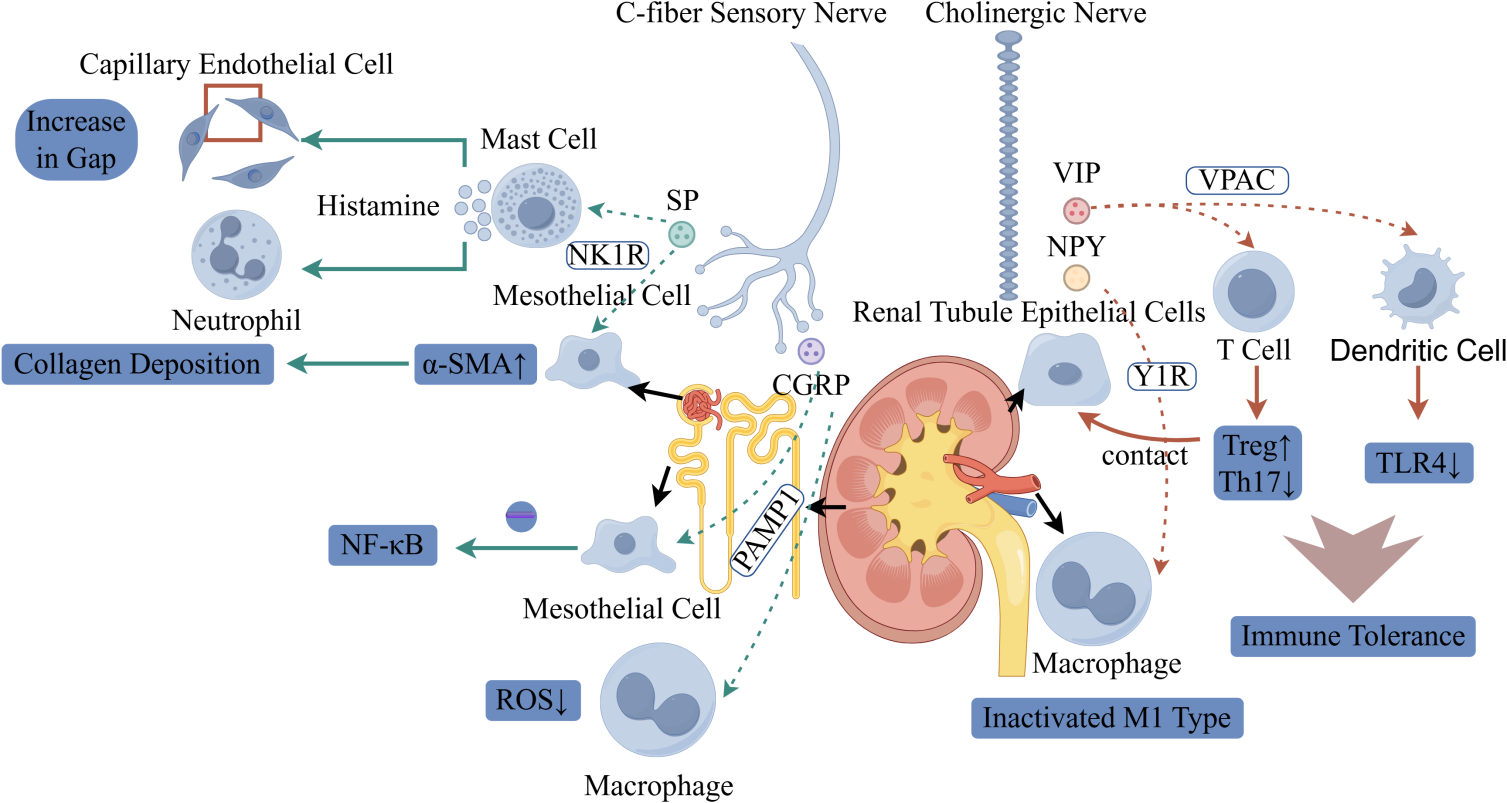
Schematic illustration of the dual regulatory roles of neural signaling in renal pathology and immunity. On the left, C-fiber sensory nerves release substance P (SP) and calcitonin gene-related peptide (CGRP): SP activates mast cells via the NK1R, triggering histamine release to induce capillary endothelial gap formation and neutrophil recruitment. SP also promotes α-smooth muscle actin (α-SMA) upregulation in mesothelial cells, driving collagen deposition (fibrosis). CGRP modulates macrophages, reducing reactive oxygen species (ROS) and regulating NF-κB signaling. On the right, cholinergic nerves secrete vasoactive intestinal peptide (VIP) and neuropeptide Y (NPY): VIP binds to VPAC receptors, while NPY signals via Y1R on renal tubule epithelial cells. Tubule-macrophage contact inactivates proinflammatory M1-type macrophages. In T cells, this neurogenic signaling promotes regulatory T cell (Treg) differentiation and suppresses Th17 cells. Dendritic cells exhibit reduced TLR4 expression, collectively enhancing immune tolerance.

The nervous system plays a pivotal bidirectional regulatory role in the renal immune microenvironment. On one hand, it suppresses excessive inflammation and maintains renal homeostasis through the release of acetylcholine and various neuropeptides. On the other hand, under pathological conditions, it can induce immune cell recruitment and promote fibrosis (17). These neuroactive substances originate not only from neurons but are also secreted by non-neuronal cells. By activating specific receptors and signaling pathways, they precisely regulate macrophage polarization, T-cell differentiation, and cytokine release. Consequently, neural mediators not only provide novel insights into the pathological mechanisms of kidney diseases but also offer potential directions for precision-targeted therapies. (The specific mechanism is illustrated in **Figures 2**, **3**).

### 3.1 Acetylcholine

The sources of acetylcholine (ACh) in RCC microenvironment exhibit marked duality. From a neuronal perspective, it is primarily released by parasympathetic nerve terminals and can remotely regulate tumor immunity via the vagus nerve-spleen axis (18). In RCC patients, vagal nerve density is significantly reduced in tumor tissues compared to adjacent normal kidney (0.8 vs 3.2 nerves/mm², p < 0.001). Orthotopic 786-O RCC xenograft models show that electrical vagus nerve stimulation (10 Hz, 5V) reduces tumor volume by 40% through enhanced ACh release and M1 macrophage infiltration (19). Meanwhile, non-neuronal sources should not be overlooked: RCC cells show dramatically decreased ChAT expression (70-80% reduction vs normal tubular cells), with lower levels correlating with higher Fuhrman grade (r = -0.68, p < 0.001). *In vitro* studies using 786-O, ACHN, and Caki-1 cell lines reveal that exogenous ACh (10-100 μM) inhibits proliferation by 40-50% and migration by 60% through α7nAChR (20). Additionally, certain macrophage populations (e.g., CD68+ macrophages) possess the capacity to secrete ACh, thereby establishing a localized immunomodulatory network, with tumor-associated macrophages (TAMs) in RCC showing reduced ACh synthesis capacity compared to normal kidney macrophages, as measured in isolated TAMs from patient samples (21) The primary action of ACh depends on the α7 nicotinic acetylcholine receptor (α7nAChR), which suppresses the expression of pro-inflammatory cytokines such as TNF-α and IL-6 by inhibiting the NF-κB pathway, while simultaneously activating the JAK2-STAT3 signaling cascade to promote the release of anti-inflammatory factors like IL-10 (12). In RCC tissues, α7nAChR is downregulated 3-fold, and its pharmacological activation with RCC xenograft growth by and enhances CD8+ T cell infiltration (22). In terms of immune cell regulation, ACh not only induces the polarization of macrophages from the M1 to the M2 phenotype but also inhibits Th17 differentiation in T cells and enhances the function of regulatory T cells (Tregs) (23, 24). In RCC microenvironment, ACh paradoxically promotes anti-tumor M1 polarization, enhances CD8+ T cell cytotoxicity against RCC cells, while RCC-infiltrating Tregs show reduced ACh responsiveness (lower STAT3 phosphorylation) compared to peripheral blood Tregs (23, 25). (**Table 1**).

**Table 1:** Signaling pathways and immunological effects mediated by key targets.

Target	Signaling pathway	Immunological effect
α7nAChR	NF-κB inhibition	TNF-α↓, IL-6↓
α7nAChR	JAK2-STAT3 activation	IL-10↑, M2 macrophage polarization↑
Renal tubular epithelial cell ACh	Autocrine ChAT pathway	Local sodium excretion↑, inflammation attenuation

### 3.2 Neuropeptides

Neuropeptides are released from sensory and autonomic nerve terminals, forming a functionally antagonistic regulatory network in RCC tumor microenvironment. For instance, substance P (SP), secreted by sensory C-fibers, exhibits significant pro-inflammatory effects (26) is elevated fold in RCC tissues and promotes tumor angiogenesis, with SP-positive nerve fibers co-localizing with CD31+ vessels in most of RCC samples (27); CGRP (calcitonin gene-related peptide), also derived from sensory nerve terminals (28), shows paradoxically increased expression in well-differentiated RCC correlating with better prognosis possibly through anti-angiogenic effects demonstrated in HUVEC-RCC co-culture assays (29); and VIP, released by autonomic nerves, possesses anti-inflammatory and immune resistance properties (30). Regarding their mechanisms of action and receptor pathways: SP activates mast cells via the NK1R pathway, promoting histamine release, increasing vascular permeability and neutrophil infiltration, and stimulating renal fibroblast proliferation. CGRP suppresses reactive oxygen species (ROS) production in macrophages through the RAMP1 pathway, mitigating oxidative stress and modulating mesangial cell inflammation. VIP induces Treg differentiation via the VPAC pathway, suppressing Th1/Th17 responses and TLR4 expression, thereby inhibiting innate immunity activation. Distinct neuropeptide receptors exhibit varied functions: The SP/NK1R axis predominantly drives pro-inflammatory and fibrotic processes. CGRP/RAMP1 exerts both antioxidant and anti-inflammatory effects, emerging as a potential protective target in acute kidney damage (AKI). VIP/VPAC1/2 promotes Treg generation and suppresses Th17 activity, demonstrating therapeutic potential in autoimmune nephropathy. NPY/Y1R inactivates M1 macrophages, offering a novel intervention strategy for AKI treatment. (**Table 2**).

**Table 2:** The major neuropeptides involved in renal pathophysiology.

Neuropeptide	Receptor	Primary effects	Pathological role
Substance P	NK1R	Pro-inflammatory, promotes fibrosis	Renal fibrosis promoter
CGRP	RAMP1	Antioxidant, bidirectional inflammatory regulation	AKI protective agent
VIP	VPAC1/VPAC2	Promotes Treg differentiation, inhibits Th17	Autoimmune nephropathy suppressor
NPY	Y1R	Enhances M1 macrophage inactivation	Novel therapeutic target for AKI

### 3.3 Norepinephrine

The primary source of norepinephrine (NE) in the RCC immune microenvironment is sympathetic nerve terminals, where it serves as a core effector molecule of the sympathetic nervous system (31). In RCC tissues, sympathetic nerve density increases compared to normal kidney, with intratumoral NE levels elevated folds (32). Under stress or pathological conditions (e.g., cardiorenal syndrome, chronic kidney disease), local NE levels in the kidney are significantly elevated (33). Patient-derived RCC xenografts (PDX) treated with propranolol (10 mg/kg) show reduced tumor growth and decreased lung metastasis (34). Notably, certain immune cells (e.g., specific macrophage subsets) may also acquire the capacity to synthesize NE by expressing enzymes such as tyrosine hydroxylase, with RCC-associated M2 macrophages showing increased TH expression and NE production capacity (35). NE exerts complex effects in the renal immune microenvironment primarily through the activation of adrenergic receptors (ARs) (36). α1-AR is overexpressed in RCC cells and promotes proliferation through MAPK/ERK signaling. Phenoxybenzamine (α-blocker, 10 μM) induces growth inhibition in 786-O and ACHN cells. *In vivo*, doxazosin treatment reduces RCC xenograft volume (37, 38). However, excessive or sustained β2-AR stimulation may also lead to excessive immunosuppression or receptor desensitization. In RCC, β2-AR activation paradoxically promotes tumor progression by inducing VEGF secretion and suppressing CD8+ T cell function. β2-AR expression on RCC-infiltrating CD8+ T cells correlates with exhaustion markers (39). ICI-118,551 (β2-AR antagonist) enhances anti-PD-1 therapy efficacy by in murine RCC models (40).

### 3.4 Dopamine

In RCC, Amino acid decarboxylase (AADC) expression is aberrantly increased in tumor cells compared to normal tubular cells, particularly in clear cell RCC with VHL mutations. Plasma and urine DA levels are elevated fold in RCC patients and correlate with tumor burden (41–43) Subsequently, DA exerts pro-tumorigenic effects in an autocrine, paracrine, or luminal endocrine manner. *In vitro*, DA enhances RCC cell proliferation and resistance to sunitinib through D2R activation. DA-treated 786-O cells show increased HIF-2α stabilization even under normoxic conditions (44). DA primarily functions through the activation of dopamine receptors (D1R–D5R), with its effects exhibiting complexity in the renal immune microenvironment. D1R expression is reduced in RCC tissues, and D1R agonist fenoldopam inhibits RCC xenograft growth while enhancing CD8+ T cell infiltration (45). Fenoldopam treatment reduces MDSC accumulation in tumors (46). In contrast, D2-like receptors (D2R, D3R, D4R) exhibit a notable bidirectional effect: low-dose activation may confer certain anti-inflammatory benefits, whereas high-dose or sustained activation tends to promote inflammation and fibrosis. For instance, through the inhibition of adenylate cyclase or activation of β-arrestin signaling, these receptors influence macrophage polarization and fibroblast proliferation (47). Consequently, dysregulation of local DA production or signaling pathways is closely associated with the pathophysiology of various kidney diseases.

### 3.5 Adenosine

Adenosine primarily functions as a critical “danger signal” in the renal immune microenvironment. It is derived from the hydrolysis of extracellular ATP/ADP catalyzed by the CD39/CD73 enzyme system. In RCC, CD73 expression is upregulated on tumor cells and correlates with advanced stage and poor prognosis (48). Hypoxic conditions in RCC further induce CD73 expression fold through HIF-2α. CD73-high RCC tumors show increased Treg infiltration and reduced CD8+ T cell function (49). RCC-derived exosomes are enriched in CD73 and create an adenosine-rich immunosuppressive niche with adenosine levels in tumor microenvironment (50). Adenosine exerts broad biological effects by activating four G protein-coupled receptors (GPCRs) — A1R, A2AR, A2BR, and A3R — with its predominant role in the immune microenvironment being immunosuppressive regulation. A2AR is highly expressed on RCC-infiltrating T cells and mediates their exhaustion. A2AR blockade with CPI-444 enhances anti-PD-1 therapy efficacy in RENCA models, increasing complete response rate. In human RCC samples, high adenosine levels correlate with M2 macrophage polarization and poor cytotoxic T cell infiltration (49, 51). Clinical trials combining A2AR antagonist CPI-444 with atezolizumab in advanced RCC show 35% objective response rate and median PFS of 6.5 months (49). Responders show reduced adenosine signature genes and increased T cell infiltration in post-treatment biopsies (52).

### 3.6 Serotonin (5-Hydroxytryptamine, 5-HT)

In RCC, intratumoral platelet accumulation is increased, with corresponding elevation of local 5-HT levels. Platelet-derived 5-HT promotes RCC progression, as platelet depletion reduces tumor growth in xenograft models (53, 54). RCC cells themselves express TPH1, particularly in metastatic lesions. TPH1 inhibition with telotristat reduces RCC growth and decreases lung metastasis (55). 5-HT exerts its biological effects through multiple receptors (at least 7 major classes and 14 subtypes), predominantly exhibiting pro-inflammatory and pro-fibrotic pathological effects in the renal immune microenvironment. 5-HT2AR is overexpressed in RCC and promotes tumor cell proliferation, migration, and VEGF secretion. 5-HT2AR antagonist ketanserin reduces RCC xenograft growth and decreases microvessel density (56).

## 4 The role of the neuroimmune system in RCC

Renal cell carcinoma (RCC), the most common malignant tumor in adults, exhibits a rising occurrence rate annually, with 80% of cases being ccRCC. Numerous clinicopathological studies have confirmed that increased neural density within and surrounding the tumor serves as a critical marker for renal tumor progression. In ccRCC, high-density sympathetic nerve fiber infiltration is significantly associated with advanced clinical stage, high ISUP grade, and distant metastasis (9). Within the TME of renal tumors, the interaction between nerves and the immune system constitutes a central driving network for disease progression.

### 4.1 The impact of nerves and their injury-induced proliferation on tumors and its relationship with immune regulation

Chronic stress or direct tumor infiltration can activate the sympathetic nervous system, leading to the release of NE. NE acts on β-adrenergic receptors expressed on TAM and tumor-infiltrating lymphocytes, promoting the secretion of factors such as vascular endothelial growth factor (VEGF) and IL-6, while suppressing CD8^+^ T cell function and inducing M2 macrophage polarization. These effects accelerate tumor angiogenesis and metastasis (57–59).

Signals of cancerous or immune origin — including growth factors, cytokines (e.g., IL-1β, IL-6, IL-10, MIP, TNF), and immunoglobulins — can sensitize nociceptive nerves, leading to neuronal hyperexcitability and the secretion of diverse neuropeptides such as substance P (SP) and calcitonin gene-related peptide (CGRP). These neuropeptides directly inhibit dendritic cell maturation, reduce antigen-presentation efficiency, and maintain an immune-resistant state by activating Tregs. Consequently, they support cancer cell proliferation, suppress tumor-associated proinflammatory cytokines, induce neovascularization, promote metastasis, modulate antigen flow in lymphatic vessels, and drive immune evasion and exhaustion (57–60).

In contrast, vagus nerve activation exhibits potential antitumor effects. The vagus nerve releases acetylcholine, which acts on cholinergic receptors on immune cells, activating relevant signaling pathways. This process may induce the activation and proliferation of NK cells and cytotoxic T lymphocytes, enhancing their ability to recognize and eliminate tumor cells. Additionally, it modulates TAM polarization, promoting their transition toward the antitumor M1 phenotype, which secretes proinflammatory cytokines such as IL-12 and TNF-α, thereby activating the host’s antitumor immune response (12).

When tumors invade nerve-deficient luminal structures, nerve injury activates Schwann cells and intrinsic nerve regeneration and repair programs. Activated Schwann cells adopt a reparative phenotype, creating pathways for cancer cells to migrate along nerve fibers. Concurrently, demyelination triggers a nerve injury response, releasing cytokines including IL-1, IL-6, IL-10, and TGF-β. The downstream immune response entails the recruitment of growth factor-secreting macrophages — including those that produce glial cell line-derived neurotrophic factor (GDNF). These macrophages not only promote cancer cell migration along nerves but also remodel the extracellular matrix (ECM), thereby facilitating neural invasion. Subsequently, this unique state of tumor-associated nerve infiltration induces distinct immune responses within the neural niche, exerting a negative impact on the overall tumor immune microenvironment and promoting regulatory and immunosuppressive responses (57–59).

TAMs secrete neurotrophic factors such as nerve growth factor (NGF) and brain-derived neurotrophic factor (BDNF), promoting abnormal nerve fiber proliferation within tumors and forming “neural invasion hotspots,” which are associated with pain and poor prognosis.

Mesencephalic astrocyte-derived neurotrophic factor (MANF) is aberrantly overexpressed in RCC. Upon MANF knockdown, phosphorylated inositol-requiring enzyme 1α (IRE1α) and the molecular chaperone binding immunoglobulin protein (BiP) are significantly upregulated, accompanied by typical stress-induced morphological changes such as endoplasmic reticulum (ER) lumen dilation. MANF deficiency triggers ER stress in RCC cells, suppressing tumor cell proliferation and invasion via the IRE1α pathway (9).

### 4.2 The impact of neuro-immune crosstalk on systemic and tumor microenvironments

Tumor-derived IL-6 disrupts the blood-brain barrier and enters the central nervous system, activating the area postrema (AP)-parabrachial nucleus (PBN) pathway. This suppresses dopaminergic neurons projecting from the substantia nigra pars reticulata (SNpr) to the ventral tegmental area (VTA), leading to functional inhibition of the nucleus accumbens (NAc) reward circuit. Consequently, this mediates disturbances in the “gut-kidney-brain axis,” contributing to cachexia-related motivational deficits. Additionally, tumor glycolysis generates excessive lactate, which promotes Treg expansion and M2 macrophage polarization via histone lactylation, thereby establishing an immunosuppressive niche (58).

Tregs regulate angiogenesis through neuronal nitric oxide synthase (nNOS). In ccRCC, nNOS is downregulated, resulting in reduced NO synthesis. This stabilizes HIF-1α, enhances VEGF pathway activity, and accelerates tumor angiogenesis. Clinically, patients with low nNOS expression exhibit significantly shorter disease-free survival (61). Under hypoxic conditions, tumor cells stabilize HIF-2α, upregulating VEGF expression while suppressing T-cell infiltration (62).

The hypoxic and lactate-rich microenvironment in RCC facilitates aberrant lysine lactylation. Specifically, lactylation at the K82 site of the YTHDC1 protein enhances its phase separation capacity, forming nuclear condensates that protect oncogenic transcripts (e.g., BCL2, E2F2) and promote tumor progression (63).

Single-cell sequencing analysis demonstrated that CD68^+^ microglia are significantly increased in the tumor microenvironment (TME) of early-stage ccRCC (TNM stage I). These microglia highly express HSPA1A/DNAJB1 heat shock proteins, while CD1c^+^ B dendritic cell (B-DC) infiltration is impaired. Consequently, CD8^+^ T cells undergo extensive exhaustion due to insufficient antigen presentation. Clinical samples confirmed an inverse correlation between microglial density and CD1c^+^ B-DC infiltration, a phenomenon exclusive to early-stage tumors. The driving factors behind microglial accumulation and their interaction with classical neural innervation require further investigation. Targeting microglial activation or restoring dendritic cell infiltration may represent novel immunotherapeutic strategies for early-stage RCC (64).

## 5 Neuroimmunology in therapeutic strategies for RCC

### 5.1 Targeted therapy of neural signaling pathways

#### 5.1.1 Adrenergic signaling blockade

Given the pivotal role of β-adrenergic receptors (β-ARs) in RCC progression, β-blockers have emerged as the most clinically translatable neuromodulatory agents. Following β-blocker administration, a reduction in immunosuppressive cells within the TME and enhanced antitumor T-cell activity are observed, leading to effective suppression of tumor growth (65, 66). However, specific data on β-blocker efficacy in RCC remain insufficient. Currently, several prospective clinical trials (e.g., NCT04848519, NCT03384836) are evaluating the therapeutic efficacy of β-blockers combined with immune checkpoint blockers (ICBs) in advanced RCC.

RCC cells predominantly express β2-AR, suggesting that nonselective β-blockers (e.g., propranolol, nadolol) may outperform selective β1-blockers. Nevertheless, the cardiovascular side effects of nonselective agents limit their clinical application. To address this issue, novel tumor-targeted β2-AR antagonists (e.g., AGN-227) are under development. These agents achieve RCC-specific accumulation through peptide modification, significantly improving therapeutic safety.

#### 5.1.2 Sensory neuromodulation

To counteract sensory nerve-mediated tumor progression and pain, multiple targeted strategies have entered clinical evaluation. Anti-NGF antibodies (e.g., tanezumab) demonstrated significant efficacy in alleviating bone metastasis-related pain in Phase III clinical trials, while potentially suppressing tumor growth via blockade of the NGF-TrkA signaling axis. Additionally, the high-affinity capsaicin analog resiniferatoxin (RTX) selectively ablates sensory nerve fibers. Renal cancer ablation and denervation have demonstrated local control effects on early renal cancer progression by improving renal hemodynamics and indirectly influencing the TME. This mechanism may involve sensory nerve inhibition, suggesting therapeutic potential for selective sensory nerve fiber ablation in the future. Furthermore, PTX has been demonstrated to inhibit IL-2 toxicity while preserving anti-tumor efficacy in patients with metastatic renal cell carcinoma. Moreover, PTX has been shown to inhibit IL-2 toxicity, preserve anti-tumor efficacy in patients with metastatic RCC (67). PTX and 5-aza-2′-deoxycytidine use lymphoid enhancer-binding factor 1 and the Wnt/β-catenin pathway to synergistically interact against RCC (68).At the molecular targeting level, neurokinin-1 receptor (NK1R) antagonists (e.g., aprepitant) and calcitonin gene-related peptide (CGRP) receptor antagonists (e.g., erenumab) have also demonstrated antitumor potential. Particularly in RCC subtypes with prominent neural infiltration, these agents may serve as adjuncts to standard therapy.

### 5.2 Remodeling of the neuroimmune microenvironment

#### 5.2.1 Combination with immune checkpoint blockers

The combined application of neural modulation and immunotherapy has emerged as a novel paradigm in RCC treatment. During RCC progression, aberrant activation of the VEGF pathway not only drives tumor angiogenesis but also shapes an inhospitable environment through multifaceted mechanisms (69, 70). Sympathetic nerve activation suppresses CD8+ T cell infiltration into tumor tissues and promotes T cell exhaustion. β-blockers can reverse this immunosuppressive state and enhance the efficacy of PD-1 inhibitors. Preclinical studies in murine models have demonstrated that anti-PD-1 antibodies promote T cell infiltration into tumor tissues while upregulating the expression of pro-inflammatory cytokines such as TNF-α, thereby significantly augmenting local immune responses (71). Anti-VEGFR-2 antibodies do not interfere with PD-1 blockade-induced T cell infiltration and immune activation. VEGFR tyrosine kinase inhibitors (TKIs) further enhance T cell recruitment to tumors by promoting vascular normalization and increasing the expression of chemoattractants such as CXCL10/11 (71). Multiple pivotal international studies have confirmed the superior efficacy of combination targeted-immunotherapy over monotherapy, particularly the RENOTORCH trial, which demonstrated that axitinib combined with toripalimab significantly prolonged progression-free survival (PFS) to 18 months with an objective response rate (ORR) of 56.7% (72, 73).

Based on the spatiotemporal dynamics of neuro-immune crosstalk, sequential therapeutic strategies may further optimize outcomes. Preclinical evidence suggests that administering β-blockers first to alleviate immunosuppression, followed by immune checkpoint blockers, more effectively activates antitumor immune responses compared to concurrent treatment (74). This finding provides a critical rationale for designing sequential neuroimmunomodulatory regimens.

#### 5.2.2 Neural regulation of tumor-associated macrophages

Single-cell studies in RCC have identified a C1QC+ TAM subset that interacts with nerve fibers via the CXCL12-CXCR4 axis, fostering an inhospitable environment. Targeting this pathway, small-molecule CXCR4 antagonists effectively disrupt neural-TAM communication and restore antitumor immunity. The synergistic effect is further enhanced when combined with CSF-1R inhibitors to block TAM recruitment (75).

Estrogen signaling also modulates the neuroimmune microenvironment in female patients with tuberous sclerosis complex-associated angiomyolipoma (TSC-AML). Selective estrogen receptor modulators (SERMs) or aromatase inhibitors suppress ERα signaling, reducing C1QC+ TAM infiltration and downregulating CXCL12 expression, thereby disrupting the neural-TAM interaction network. This sex-specific therapeutic strategy offers a precision intervention for female RCC patients (75).

### 5.3 Nerve blockade and ablation techniques

For RCC with exceptionally high neural density, local nerve blockade demonstrates unique therapeutic value. Percutaneous image-guided radiofrequency ablation (RFA) of the perirenal nerve plexus selectively disrupts tumor-associated nerve fibers via thermal effects. Clinical studies indicate that this technique not only alleviates tumor-related pain but also directly inhibits tumor growth and enhances the efficacy of targeted therapies (76).

At the renal hilum, chemical nerve blockade can more extensively interrupt the renal-brain sympathetic circuit. In RCC patients with refractory hypertension, this approach simultaneously controls tumor progression and blood pressure, thereby improving cardiac and renal function (16).

### 5.4 Emerging targets and intervention strategies

In **Table 3**, we list some ongoing clinical trials for the treatment of renal cell carcinoma. They are expected to achieve good results in the treatment of RCC.

**Table 3:** The ongoing clinical trials related to RCC treatment.

Intervention	Trial design	Patient group	Primary outcomes	NCT number
Cabozantinib + Nivolumab	Phase III, Randomized, Double-blind	Previously untreated intermediate-/poor-risk advanced RCC	PFS by BICR	NCT03937219
Avelumab + Axitinib	Phase III, Randomized	Previously untreated advanced RCC (all IMDC risk groups)	OS, PFS in PD-L1+ and overall population	NCT02684006
Pembrolizumab + Cabozantinib	Phase I/II	Metastatic RCC (clear cell or non-clear cell)	ORR, Safety	NCT03149822
Personalized neoantigen vaccine	Phase 1	Advanced ccRCC	Immune activation,RFS	NCT02950766

ORR, objective response rate; PFS, progression-free survival; OS, overall survival; BICR: blinded independent central review.

#### 5.4.1 Targeting the ALKBH5-MANF-ER stress axis

To address ER stress resistance mechanisms in RCC, ALKBH5 small-molecule inhibitors have been demonstrated to effectively reduce MANF expression in preclinical models, thereby enhancing tumor cell susceptibility to ER stress-induced apoptosis. When combined with VEGFR inhibitors (e.g., cabozantinib), this approach significantly delays disease progression in VHL-deficient RCC (9, 76).

Furthermore, targeting the IRE1α-XBP1 pathway—such as with the IRE1α inhibitor KIRA6—can overcome tumor ER stress resistance, offering a complementary therapeutic strategy.

#### 5.4.2 Neurotransmitter-targeted delivery systems

For brain-metastatic RCC, GABA receptor modulation has emerged as a promising therapeutic direction. Baclofen, a GABAB receptor agonist, not only suppresses the growth of RCC brain metastases but also reduces seizure frequency by modulating tumor-associated neuronal activity, thereby improving patients’ quality of life (60).

#### 5.4.3 Personalized neoantigen vaccines

Recent studies have revealed that neoantigens, derived from tumor-specific mutations, serve as pivotal targets in antitumor immunity and play a critical role in activating host immune responses against cancer. Personalized cancer vaccines (PCVs), an innovative therapeutic approach based on neoantigen profiling, precisely identify patient-specific tumor signatures and induce immune responses against cancer-specific epitopes. A recent phase 1 clinical trial involving nine patients with advanced ccRCC demonstrated that PCVs triggered immune activation within just three weeks, with all patients remaining recurrence-free for three years (77).

## 6 Prospects and conclusions

Research on the neuroimmune microenvironment of RCC is advancing at an unprecedented pace, yet this emerging field still faces multiple challenges. The spatiotemporal-specific roles of different neural subtypes (sympathetic, parasympathetic, and sensory nerves) in tumor progression remain unclear. Furthermore, the dynamic crosstalk between neural, immune, and tumor components—along with its influence on therapeutic responses — requires further investigation. A critical unresolved challenge lies in achieving spatiotemporal precision in neural-targeted interventions while mitigating systemic side effects.

Key future research directions include: (1) Leveraging single-cell multi-omics combined with spatial transcriptomics to delineate a high-resolution atlas of the neuroimmune microenvironment in RCC; (2) Establishing a precision classification system guided by neuroimmune biomarkers; (3) Developing tumor-targeted delivery systems for neuroactive drugs to enhance therapeutic indices; (4) Exploring optimal combination strategies and sequencing schedules between neuromodulation and existing therapies.

The application of neuroimmune regulation in RCC treatment may extend beyond tumor control. For instance, interventions targeting novel antidiuretic pathways (e.g., the ITPF-NK3R axis) in tumor-associated renal dysfunction could not only improve renal function but also suppress tumor progression by disrupting the kidney-brain reflex arc. Similarly, targeting sympathetic reflex circuits involving the kidney-brain-vascular/adipose axis in RCC patients with common comorbidities such as hypertension and insulin resistance may achieve multi-organ protective effects with a single therapeutic approach.

As a core component of the renal tumor microenvironment, the neuroimmune network is reshaping our understanding of RCC pathogenesis and providing novel therapeutic targets and strategies. With deeper integration of basic and clinical research, interventions targeting the neuroimmune microenvironment are poised to become potential new directions of comprehensive RCC treatment, following anti-angiogenic therapy and immunotherapy, ultimately improving patient survival and quality of life.

## Glossary

RCC: Renal cell carcinoma
TME: Tumor microenvironment
TAM: Tumor-associated macrophage
ccRCC: Clear cell renal cell carcinoma
ADRB2: β2-adrenergic receptors
TNTs: Tunneling nanotubes
NE: Norepinephrine
NETs: Norepinephrine transporters
Tregs: Regulatory T cells
NK1R: Neurokinin 1 receptor
DC: Dendritic cell
TH: Tyrosine hydroxylase
IML: Intermediolateral
DRG: Dorsal root ganglion
ATP: Adenosine triphosphate
NPY: Neuropeptide Y
VIP: Vasoactive intestinal peptide
SGLT2: Sodium-glucose cotransporter 2
CNS: Central nervous system
PVN: Paraventricular nucleus
CKD: Chronic kidney disease
RAAS: Renin-angiotensin-aldosterone system
Ach: Acetylcholine
ChAT: Choline acetyltransferase
α7nAChR: α7 nicotinic acetylcholine receptor
SP: Substance P
ROS: Reactive oxygen species
AKI: Acute kidney damage
ARs: Adrenergic receptors
DA: Dopamine
AADC: Amino acid decarboxylase
GPCRs: G protein-coupled receptors
GFR: Glomerular filtration rate
5-HT: 5-Hydroxytryptamine
TPH1: Tryptophan hydroxylase 1
5-HT2AR: 5-HT2A receptor
EMT: Epithelial-mesenchymal transition
eCBs: Endocannabinoids
AEA: Aanandamide
2-AG: 2-arachidonoylglycerol
VEGF: Vascular endothelial growth factor
GDNF: Glial cell line-derived neurotrophic factor
NGF: Nerve growth factor
BDNF: Brain-derived neurotrophic factor
MANF: Mesencephalic astrocyte-derived neurotrophic factor
IRE1α: Inositol-requiring enzyme 1α
BiP: Binding immunoglobulin protein
ER: Endoplasmic reticulum
AP: Area postrema
PBN: Parabrachial nucleus
SNpr: Substantia nigra pars reticulata
VTA: Ventral tegmental area
NAc: Nucleus accumbens
nNOS: Neuronal nitric oxide synthase
B-DC: B dendritic cell
β-ARs: β-adrenergic receptors
ICBs: Immune checkpoint blockers
RTX: Resiniferatoxin
NK1R: Neurokinin-1 receptor
CGRP: Calcitonin gene-related peptide
TKIs: Tyrosine kinase inhibitors
PFS: Progression-free survival
ORR: Objective response rate
TSC-AML: Tuberous sclerosis complex-associated angiomyolipoma
SERMs: Selective estrogen receptor modulators
RFA: Radiofrequency ablation
PCVs: Personalized cancer vaccines
